# Why Does Obesity as an Inflammatory Condition Predispose to Colorectal Cancer?

**DOI:** 10.3390/jcm12072451

**Published:** 2023-03-23

**Authors:** Anna Maria Rychter, Liliana Łykowska-Szuber, Agnieszka Zawada, Aleksandra Szymczak-Tomczak, Alicja Ewa Ratajczak, Kinga Skoracka, Michalina Kolan, Agnieszka Dobrowolska, Iwona Krela-Kaźmierczak

**Affiliations:** 1 Department of Gastroenterology, Dietetics and Internal Diseases, Poznan University of Medical Sciences, Przybyszewskiego 49, 60-355 Poznan, Poland; 2 Doctoral School, Poznan University of Medical Sciences, Bukowska 70, 60-812 Poznan, Poland; 3 Faculty of Medicine Ludwik Rydygier Collegium Medicum, Nicolaus Copernicus University, 85-094 Bydgoszcz, Poland

**Keywords:** obesity, inflammation, colorectal cancer, diet, gut microbiota

## Abstract

Obesity is a complex and multifactorial problem of global importance. Additionally, obesity causes chronic inflammation, upregulates cell growth, disturbs the immune system, and causes genomic instability, increasing the risk of carcinogenesis. Colorectal cancer is one of the most common cancers, and it has become a global problem. In 2018, there were around 1.8 million new cases and around 881,000 deaths worldwide. Another risk factor of colorectal cancer associated with obesity is poor diet. A Western diet, including a high intake of red and processed meat and a low consumption of whole grains, fruits, vegetables, and fiber, may increase the risk of both colorectal cancer and obesity. Moreover, the Western diet is associated with a proinflammatory profile diet, which may also affect chronic low-grade inflammation. In fact, people with obesity often present gut dysbiosis, increased inflammation, and risk of colorectal cancer. In this article, the association between obesity and colorectal cancer is discussed, including the most important mechanisms, such as low-grade chronic inflammation, gut dysbiosis, and poor diet.

## 1. Introduction

The overall number of people with obesity worldwide has tripled compared to 1975. According to available data, in 2016, more than 1.9 billion adults were overweight (39% of the adult population), and 650 million had obesity (13% of the population). Obesity is an essential factor affecting cancerogenesis. It is linked to cancer development by several mechanisms, e.g., by sustaining proliferative signaling, increasing tumor-promoting inflammatory cytokines, affecting immune functions, upregulating cell growth, and/or suppressing cell death. Moreover, obesity is a disease linked to chronic low-grade inflammation associated with increased genomic instability [1].

Further, colorectal cancer (CRC) is the third most common cancer worldwide. It is estimated that nearly two million patients were diagnosed with this type of cancer in 2020. Approximately one million people die annually from colorectal cancer [2]. The incidence of CRC is associated with numerous modifiable and non-modifiable factors, most of which have been well studied and understood [3]. Non-modifiable factors include age and hereditary factors. Modifiable factors include diet and lifestyle factors, such as low physical activity, being overweight (BMI (kg/m^2^) in the ranges of 25–29.9) or having obesity (BMI ≥ 30 kg/m^2^), poor diet (including excessive red meat consumption, low dietary fiber intake, and alcohol drinking), and smoking [4].

In recent years, reports confirming the link between inflammation and cancer development have increased [5,6]. According to the World Health Organization (WHO) and the International Agency for Research on Cancer (IARC), both obesity and colorectal cancer are significant health problems in an increasingly young population as well.

Researchers are particularly interested in the link between obesity and the incidence of colorectal cancer [7]. Chronic inflammation has recently been shown to be a key factor predisposing towards CRC development [8], and several reviews can be found on this topic. For example, a review by Malhab et al. focused on the molecular level of the CRC–obesity relationship [9]. Immunological signaling pathways in the inflamed intestinal mucosa, which include nuclear factor (NF)-κB, prostaglandin E2 (PGE2)/cyclooxygenase-2 (COX-2), interleukin (IL)-6/signal transducer and activator of transcription 3 (STAT3), and IL-23/T helper 17 cells (Th17), lead to tumorigenesis. In addition, reactive oxygen species derived directly from inflammatory cells can interfere with carcinogenic genes, such as p53. On the other hand, a review by Vazzana et al. also focused on adipose tissue and chosen adipokines [10]. Adipose tissue functions as an endocrine organ that regulates the body’s energy and metabolic homeostasis. It has been suggested that excess adipose tissue promotes the secretion of numerous adipokines, cytokines, and free radicals. Thus, potential mechanisms underlying the association of obesity with CRC pathogenesis include adipocytokine imbalance, insulin resistance, changes in the insulin-like growth factor (IGF)-1/IGF-1 receptor (IGF-1R) axis, and chronic inflammation and oxidative stress [2].

However, apart from obesity itself, other obesity-related factors can also affect chronic inflammation, indirectly increasing the risk of CRC. For example, a growing body of research also highlights the link between obesity, bacterial dysbiosis, and consequent intestinal barrier dysfunction and the incidence of colorectal cancer. The development of CRC is associated with the loss of intestinal barrier integrity and the activation of proinflammatory IL-23/IL-17 signaling, leading to tumorigenesis [11]. Species associated with the development of CRC include *Fusobacterium Nucleatum*, *Streptococcus gallolyticus*, *Bacteroides fragilis*, *E. coli*, and *Enterococcus faecalis*. In individuals with obesity, the composition of the intestinal microbiome is poorer in bacteria with anti-inflammatory effects. The microbiome’s influence on inflammatory pathways leading directly to carcinogenesis has also been postulated [12]. However, in recent years, the possibility of dietary interventions aimed at influencing the gut microbiota and inflammatory pathways has also been postulated to have a potential protective effect against carcinogenesis. This could provide an additional diet-related perspective, as generally, poor diet is an important and common factor leading to both obesity and colorectal cancer. It can affect carcinogenesis in the colon through direct effects on the immune system and inflammation and indirectly through hyperalimentation, leading to overweight and obesity, which are CRC risk factors.

The purpose of this article is to introduce the reader to the vital relationship between obesity related to chronic and low-grade inflammation and the incidence of colorectal cancer and to highlight the importance of other obesity-related factors that can indirectly increase CRC risk by increasing obesity-related inflammation. Knowing and understanding how obesity leads to tumorigenesis is an essential step in developing therapies for patients with obesity and colorectal cancer.

## 2. Colorectal Cancer

### 2.1. Epidemiology of Colorectal Cancer

Colorectal cancer is one of the most common cancers. It ranks third in terms of incidence and is the second leading cause of cancer-related deaths. In 2018, there were around 1.8 million new cases and around 881,000 deaths worldwide [13]. With the continuous progress of civilization, the incidence of colorectal cancer is projected to increase to 2.2 million new cases and 1.1 million deaths worldwide by 2030 [13]. The median age of patients at diagnosis is 72 years in women and 68 years in men. For rectal cancer, the median age is 63 years for both sexes. Although colorectal cancer still ranks high, its incidence and mortality have decreased in recent years in Europe and the USA. There has been a 2–3% decline per year since 2000 [14]. The observed trend is undoubtedly associated with extensive screening programs and, thus, with the possibility of preventing cancer development by removing precancerous lesions, as well as with greater public awareness of both the detection and prevention of colorectal cancer. The 5-year survival rate in this type of cancer is 60%, considering all stages of the disease. Considering only advanced cancer with metastases, the prognosis is definitively worse. In this subgroup, 5-year survival is observed in 14% of patients [14]. It is estimated that 608,570 Americans will die of cancer in 2021, which is equivalent to more than 1600 deaths per day. The largest number of deaths are lung, prostate, and colorectal cancers in men and lung, breast, and colorectal cancers in women [14]. Although, as mentioned above, colorectal cancer is detected mainly around the age of 70, much attention has been paid in recent years to early colorectal cancer, i.e., cases that are detected before the age of 50. In 2010, the incidence this type of cancer was 4.8% for colon cancers and 9.5% for rectal cancers [15]. Although the incidence of colorectal cancer is decreasing, many studies report an increased incidence of colorectal cancer among younger patients. The research by Pan et al. on the incidence of early colorectal cancer is interesting [16]. These researchers found a 136.9% increase in cases worldwide in 2019, compared to the previous thirty years. They see the reason for this state, among others, in the increase in economic status in many regions. Behavioral changes are observed, especially in developing countries. These include a higher consumption of highly processed foods and red meat, physical inactivity, obesity, smoking, and higher alcohol consumption. This lifestyle is characteristic mainly of people born in the second half of the 20th century. More than half of the cases of colorectal cancer occur in highly developed countries [17,18].

Ma et al. in a meta-analysis of 41 studies on generalized obesity and 13 studies on central obesity, correlated these conditions with an increased risk of colorectal cancer [19]. It is estimated that around 11% of European colorectal cancer cases are related to overweight or obesity in Europe. Epidemiological data suggest that obesity is associated with a 30–70% increased risk of colorectal cancer in men; this relationship is not as high in females [20,21]. Since 2019, the diagnosis and treatment of cancer have been affected by the coronavirus (COVID-19) pandemic [22,23]. Limited access to healthcare facilities has led to cancer diagnosis and treatment delays. This will undoubtedly be associated with a temporary decrease in the incidence of cancer in the last 2 years, but also with a subsequent increase in the incidence of cancer in the advanced stage of the disease. However, we do not yet have population data on this subject.

### 2.2. Risk Factors of Colorectal Cancer

Both environmental and genetic factors play a role in the etiology of colorectal cancer. The cumulative lifetime risk of colorectal cancer in the general population ranges from 5–6%. In a quarter of patients, a potential, identifiable genetic cause can be found [24]. If a first-line family member has had colorectal cancer, the risk of developing the disease doubles and increases with the number of affected relatives—e.g., with two or more relatives affected, the risk increases even four-fold [25]. In sporadic colorectal cancer, a positive family history confirms the participation of low penetrance genetic factors [26,27]. Lynch syndrome is the most common inherited colorectal cancer syndrome. Patients covered by this syndrome account for 5–10% of all cases [28,29]. This syndrome is caused by a mutation in one of the DNA mismatch repair genes: MLH1, MSH2, MSH6, PMS2, or EPCAM [30]. The second most common hereditary colorectal cancer syndrome is Familial Polyposis Syndrome (FAP). This syndrome is associated with gene mutations that control the Wnt-signaling pathway [31].

The risk of developing colorectal cancer also increases among patients with inflammatory bowel diseases, mainly ulcerative colitis. It is estimated that 1% of colorectal cancer patients are IBD patients [32]. In recent years, there has been a decrease in the number of cases in this group of patients. This is due to improved treatment and supervision of these patients [33].

As mentioned above, the incidence of colorectal cancer is higher in highly industrialized countries [17,18]. This is related to the influence of environmental factors on the occurrence of this type of cancer. Studies in the literature have proven that 16–71% of cancer cases in Europe and the United States are colorectal cancer, which can be directly related to lifestyle [34,35].

These lifestyle factors include smoking, higher alcohol consumption, a diet rich in highly processed products, the consumption of red meat, and physical inactivity. Obesity has been shown to increase the risk of colorectal cancer by about 2–3% for every one-unit increase in BMI above normal weight [36]. It should be noted here that the dominant view in research is that the risk of colorectal cancer is higher in visceral obesity compared to subcutaneous obesity [20]. In addition, obesity is associated with poorer treatment outcomes and higher mortality. The mechanisms linking obesity and colorectal cancer are still the subject of much research [19]. Nutritional factors also may affect the risk of CRC development [37]. According to a meta-analysis, dietary fiber, especially from cereal and whole grains, decreases the risk of colorectal cancer [38]. Vitamin D also influences CRC risk. Hernández-Alonso et al. reported that circulating vitamin D correlates negatively with the risk of colorectal cancer [39]. The European Prospective Investigation into Cancer and Nutrition reported that calcium and dairy product intake is inversely associated with the risk of CRC [40]. It is vital to acknowledge that physical activity plays an important role in the prevention of CRC. The World Cancer Fund International found that physical activity decreases the risk of CRC [41]. In their study, Morris et al. reported that a higher level of physical activity was associated with a lower CRC risk [42]. Several studies have also been devoted to the preventive effect of drugs. There are reports in the literature about the beneficial effects of non-steroidal anti-inflammatory drugs, aspirin, statins, and hormone replacement therapy in postmenopausal women; however, none of these drugs have found an unquestionable place in the prevention of colorectal cancer; researchers believe that their preventive effect may be related to the patient’s genotype [43,44].

Among environmental factors in the pathogenesis of colorectal cancer, much attention in the literature is devoted to the role of intestinal microbiota. There is increasing evidence that it plays a role in both the initiation and progression of bowel cancers. The intestinal microbiome analysis showed differences in the composition between healthy people and patients with colorectal cancer [45,46]. Changes in the microbiome have also been observed in the early stages of cancer. Researchers suggest that the analysis of the microbiome’s composition may be an early marker of colorectal cancer detection; on the other hand, its appropriate modulation may reduce the risk of its formation. Further research is needed in this area [47].

## 3. Obesity and Colorectal Cancer

### 3.1. Epidemiology of Obesity as a Risk Factor for Colorectal Cancer

Obesity is a complex and multifactorial problem of global importance. According to the WHO, the population of people with obesity has tripled since 1975. Regardless of gender, age, and material status, excess body weight and its implications are a growing problem with epidemic status [2,48]. According to the WHO, every third person over 18 years old in 2016 was overweight, and 13% of the population had obesity [49,50]. Such a rapid and large increase in obesity statistics is extremely worrying, since obesity is considered an essential environmental risk factor for CRC. It is estimated that among people with obesity, the risk of CRC is 7% to up to 60% higher than among healthy individuals [51,52]. Further, according to the available data, sex-related differences can be observed when investigating obesity and CRC risk, with possibly higher risk among men with obesity than among women with obesity [51]. Sex-related differences can be multifactorial, but several behavioral factors could explain the observed differences. For example, men are more likely than women to abuse alcohol, smoke cigarettes, and follow a diet that includes large amounts of red meat and processed foods [53]. Obesity-related increased risk of CRC can depend on whether obesity is general (measured only by BMI), central (for example, waist circumference), or even on the duration of obesity.

According to the meta-analysis of Goodarzi et al., the association between obesity and increased CRC risk is irrespective of metabolic status; however, sex-related differences were also observed [54]. Among metabolically healthy obesity and metabolically unhealthy obesity, a 14% and 24% increased risk of CRC was observed, respectively; however, when gender was taken into account, the increased risk of CRC was still significant only for men. Interestingly, it should be noted that metabolic abnormality—even when body weight is normal—was associated with a 19% increased risk of CRC (OR = 1.19; 95% CI = 1.09−1.31). In a study by Li et al. BMI, waist–hip ratio (WHR), and waist circumference (WC) were associated with a significantly increased risk of colon (not rectal) cancer in men only [55]. For the highest quintile of BMI, WHR, and WC, hazard ratios (HRs) for colon cancer were 2.15, 1.97, and 2.00, respectively. On the other hand, in a study by MacInnis et al., central obesity was positively associated with the risk of colon cancer among women. In this cohort, each 5 kg/m^2^ increase in BMI insignificantly increased the risk of colon cancer by 4% (HR = 1.04 (0.90–1.20), *p* = 0.59), but each 10 cm of waist circumference significantly increased the risk of colon cancer by 14% (HR = 1.14 (1.02–1.28), *p* = 0.02) [56].

Interestingly, recent data suggest that obesity at an early age significantly affects colorectal cancer development—and its risk—in later life. In a meta-analysis by Garcia et al. [57], early-life obesity significantly increased the risk of colorectal cancer by 39% (relative risk, RR = 1.39; 95% CI = 1.20−1.62) and 19% (RR = 1.19; 95%CI = 1.06−1.35) among men and women, respectively, when compared with controls. Interestingly, in men, obesity during early life more significantly increased the risk of distal colon and rectal cancer than proximal colon cancer—by 51%, 39%, and 6%, respectively. Among women, early-life obesity increased the risk of rectal cancer more significantly than proximal and distal colon cancers—by 38% vs. 8% in both cases, respectively. Similar findings were observed in a study by Hidayat et al., in which each 5 kg/m^2^ increase in body mass index in early life (<30 years) was significantly associated with a 13% increase in colorectal cancer (RR 1.13, 95% CI 1.08, 1.19) [58].

In a meta-analysis by Ma et al., obesity significantly increased CRC risk by 34% (RR = 1.344, 95% CI:1.258–1.436) when compared with normal body weight [19]. A similar risk increase was observed for colon and rectal cancers. Further, a higher waist circumference increased the risk of CRC by 45% compared to a lower waist circumference (RR = 1.455; 95% CI, 1.327–1.596).

Obesity is undoubtedly an important factor in increasing the risk of CRC. However, the findings are not always consistent, and the magnitude of the association greatly varies. Moreover, more studies are needed to investigate observed differences between obesity and the risk of colorectal cancer and anatomic site- and sex-related variables. The summary of selected studies discussing the association between obesity and CRC risk can be seen in Table 1. However, the World Cancer Research Fund International considers overweight and obesity a CRC risk with strong evidence [41].

### 3.2. Pathogenesis of Colorectal Cancer in Obesity

Although obesity undoubtedly influences CRC risk, the exact mechanism is not fully understood. It is challenging to investigate or to transfer in vitro studies, since obesity does not act through one single pathway but rather affects systemic, metabolic, and further cellular and molecular environments.

Among others, two pathways seem especially essential in the CRC–obesity association—the insulin/insulin-like growth factor axis and PI3K/Akt. Obesity and increased content of visceral adipose tissue are associated with several metabolic changes—e.g., hyperinsulinemia, insulin resistance—or increased oxidative stress, altering the insulin–IGF axis. IGF is one of the factors associated with the proliferation and inactivation of apoptosis influencing carcinogenesis. Further, both IGF and insulin activate the PI3K/Akt pathway, leading to increased survival and cell growth, and IGF impairs p53, increasing neoplasia and cell proliferation. It also activates PI3K/Akt/mTORC, as well as Raf/MAPK signaling pathways or glucose transporters [66]. In an vitro study, hyperinsulinemia promoted the growth of colon cancer cells [67].

In a meta-analysis by Renehan et al., circulating IGF-1 concentrations were significantly associated with an increased risk of colorectal cancer. Odds ratios of CRC were 58% increased (1.58 (1.11–2.27)) when the highest and lowest IGF-1 categories were compared [68]. Moreover, a dose–response association was observed; however, it was not statistically significant. Similar findings were observed in another meta-analysis by Rinaldi et al., in which IGF-1 concentrations were modestly associated with CRC risk: each one standard deviation of IGF-1 increased the risk of CRC by 7% (RR = 1.07 (1.01–1.14) [69]. Other studies also confirm the role of IGF-1 concentrations in CRC development [70].

Moreover, adipose tissue macrophages infiltrating into visceral adipose tissue change the M1 (source of proinflammatory cytokines and oxygen radicals) macrophages-to-M2 macrophages ratio, which is an essential microenvironmental factor in obesity-induced adipose tissue inflammation [71,72]. In most tumors, macrophages seem to be switched into the M2 phenotype, providing an immunosuppressive microenvironment and promoting tumor growth, with the obesity-affected extracellular matrix as one of the promoters [73,74].

Further, upregulated secretion of adipokines (e.g., leptin or adiponectin), interleukins (e.g., IL-1, IL-6, IL-12), and proinflammatory cytokines (e.g., THF-α) are associated with low-grade inflammation, contributing to CRC. Several adipokines have been linked to CRC development by affecting signaling pathways (metabolic, inflammatory, and cell cycle) pathways. For example, in in vitro studies, leptin has been found to be an important factor for CRC development in obesity, and it should be noted that serum leptin concentrations can be as much as five times higher among individuals with obesity versus those in normal-weight individuals. Among individuals with obesity, the expression of leptin is altered, leading to increased leptin concentrations—and leptin resistance—and impaired binding to leptin receptors (binding leptin to its receptors inhibits food intake and increases energy expenditure) [75]. Although the exact mechanisms are not fully understood, it is assumed that decreased transport of leptin through the blood–brain barrier is the main cause of leptin resistance [76]. Leptin activates Src kinase and PI3K in the CRC cells LS174T and HM7, inducing activation of Rac1 and Cdc43, promoting carcinogenesis and metastasis [77]. In a study by Endo et al. increased proliferative activity of colonic cells (murine) was observed in the obesity model, and, moreover, increased expression of leptin receptors was observed in colorectal tumors [78]. On the other hand, in decreased leptin concentration conditions (leptin-deficient tumors), a decrease in tumor cell proliferation and tumor growth was observed. However, in vivo studies do not always confirm these results, possibly because of several other interactions with other cytokines or hormones in real-life conditions. For example, in the animal model study of Aparicio et al., hyperleptinemia did not induce variation in tumor volume or weight and did not modify the number, size, or distribution of intestinal adenomas [79].

On the other hand, another adipokine, adiponectin, acts like a regulator of colon epithelial cell homeostasis, but its concentrations are decreased among individuals with obesity. Although the mechanisms need further examination, it is assumed that during obesity-related inflammation, increased concentrations of various proinflammatory cytokines inhibit adiponectin transcription, but this could also be related to dyslipidemia, which negatively correlates with adiponectin levels [80,81]. An in vitro study by Fenton et al. showed that adiponectin acts on preneoplastic colon epithelial cells to regulate cell growth via two distinct pathways, inhibiting leptin-induced nuclear factor kappa B (NF-κB)-dependent autocrine IL-6 production and trans-IL-6 signaling [82]. However, in a meta-analysis by Joshi et al. adiponectin was associated with decreased CRC risk in only prospective studies (generally, it did not influence CRC risk).

All the above-mentioned factors affect PI3K/Akt signaling, reducing apoptosis, stimulating cell growth, and increasing proliferation in colon cancer cells. Linked to sustained proliferative signaling, obesity leads to downregulated functions of anti-growth factors and, moreover, the PI3K/Akt pathway is associated with disrupted growth factor receptors, e.g., *KRAS*, *PIK3CA*, and *PTEN* [83,84].

## 4. Gut Microbiota and Colorectal Cancer

In addition to the usual factors, such as insulin resistance in peripheral tissues, altered secretion of adipocytokines, or dysregulation of fatty acid synthesis, the gut microbiota plays an important role. Among individuals with obesity, the microbiota has a completely different composition than in normal-weight people. Intestinal bacteria are responsible for the breakdown of food residues through fermentation, with the simultaneous production of short-chain fatty acids (SCFAs) that provide nutrients for colon epithelial cells. The disruption of the microbiota observed in individuals with obesity, resulting from a change in the ratio of *Bacteroides* to *Firmicutes*, is not without its effects on the function of the colonic epithelium.

Bacterial dysbiosis occurring in individuals with obesity caused by an increase in *Firmicutes* and a decrease in *Bacteroides*, increased intestinal permeability, and the passage of endotoxic lipopolysaccharide (LPS) beyond the intestinal barrier with a concomitant increase in inflammation in individuals with obesity promotes the transformation of adenoma into invasive cancer [85]. In obesity, increased amounts of LPS activate the CD14 receptor [86]. This complex binds to toll-like receptor 4 (TLR4) on macrophages in adipose tissue. The formation of the signaling pathway results in the activation of the expression of genes encoding proinflammatory proteins such as NF-κB and activation protein 1 (AP-1). The administration of *Lactobacillus* upregulates the expression of the TLR4 and TLR2 receptor, influencing the incidence not only of obesity but also of colon cancer [87]. Higher blood levels of LPS were found in those with villous rather than tubular adenomas [88]. It is also known that a higher BMI is associated with higher levels of LPS in the blood, and a restrictive diet reduces LPS levels [89].

Further, a study by Wilson et al. showed that high fiber intake affects colon epithelial wellbeing and prevents colon cancer [90]. Fiber supplementation increased SCFAs, including butyrate, in addition to increasing the normal proliferation of healthy colonocytes. Butyrate causes strengthening of the intestinal barrier and stimulates the synthesis of anti-inflammatory cytokines (interleukin 10), and it inhibits the activation of nuclear transcription factor kappa B. In addition, it affects the immunogenicity of cancer cells by regulating the activity of proteins involved in apoptosis (Bcl-2, caspase 3, caspase 7) and increasing antioxidant activity [91,92]. Prebiotics also play an important role in preventing intestinal cancer by preventing obesity. In a study by Nicolucci, the author observed a significant increase in *Bifidobacterium* in children taking inulin fructans. In addition, there was a reduction in total body fat, no excessive weight gain, and a reduction in primary bile acids in the stool. Di-Wei Zheng posited that the use of probiotics and prebiotics can favorably affect the course of colon cancer [93]. In addition, the authors increased the abundance of various SCFA-producing bacteria, including, for example, *Eubacterium* and *Roseburia* [94]. In addition to strains typically associated with colorectal cancer risk, such as *Fusobaterium nucleatum*, patients with this condition have an imbalance of other species in favor of *Enterococcus faecalis*, *Escherichia coli*, or *Bacteroides fragilis* and a deficiency of basic strains such as *Clostridiales*, *Lactobacillus*, or *Bifidobacterium* [95,96].

Reduced bacterial diversity in obesity also promotes the development of intestinal cancer [97]. It is also known that altered gene expression and toxic effects of environmental factors play roles in the process of carcinogenesis [98]. Butyrate deficiency is also important in people with colorectal cancer [45]. Thus, in individuals with obesity, the risk of developing intestinal cancer may be increased by significantly reducing the number of SCFA-producing bacteria in the intestine [99]. The co-occurrence of diabetes and obesity increases the risk of colorectal cancer [100]. In T2DM and obesity, a significant decrease in butyrate-producing bacteria, such as *Roseburia* and *Faecalibacterium prauznitzii*, has been demonstrated [101]. Branched-chain amino acids (BCAA) are also important in exacerbating insulin resistance and obesity and disrupting insulin action [102]. A high-fat diet rich in BCAA increases the risk of developing T2DM [103]. The intestinal microbiota is one of the most important sources of BCAA. In addition, increased proteolysis of BCAA is associated with an increase in *Clostridium*. There are many studies that have looked for bacteria pathognomonic for colorectal cancer. One study found that patients with colorectal cancer have a less diverse microbiome than healthy people [46]. Some species, such as *Akkermansia* spp., *Porphyromonadaceae*, *Alistipes*, *Staphylococcaceae*, and *Methanobacteriales,* are represented more abundantly in people with colorectal cancer [45]. The amount of others, such as *Bifidobacterium*, *Lactobacillus*, *Ruminococcus*, *Faecalibacterium* spp., and *Roseburia,* was significantly reduced in those with the disorder [45]. People with colorectal cancer develop colonization of the gut with bacterial flora typical of the oral cavity [104]. In the Castelarin study, the amount of *Fusobacterium* equivocated in feces, and tumor tissue was significantly higher in intestinal tumor patients than in non-cancerous samples [105]. It was also found to be the only biomarker for non-invasive screening of colorectal cancer, and colorectal adenoma and adenocarcinoma *Fusobacterium* nucleatum exerts its carcinogenic effects by increasing cancer cell proliferation and migration and induces Cdk5 expression and activation of the Wnt-/β-catenin-signaling pathway [106]. On the other hand, the main drug used in the pre-diabetic state of insulin resistance, type 2 diabetes, and obesity—metformin—inhibits colorectal cancer precisely by affecting the mechanism of action of *Fusobacterium nucleatum* [107]. The role of *Akkermansia muciniphila* in the process of carcinogenesis in the large intestine is ambiguous; a deficiency in this bacterium is associated with the occurrence of obesity, as well as higher levels of triglycerides and blood glucose [108]. Colorectal cancer patients also showed an increase in *E. coli* [109]. The amount of mucosa-associated and internalized *E. coli* is associated with the cell proliferation index, as assessed by Ki-67 expression [110].

Fecal bacteria present in excess in the intestine secrete enzymes such as nitroreductase, glucuronidase, and azoreductase, which catalyze the release of pro-cancerogenic substances in the intestine [111]. It has been observed that there is a decrease in the activity of these enzymes in feces after supplementation with *Lactobacillus acidophilus* in both animal and human models [111]. In addition, it is also emphasized that the more frequent occurrence of colon cancer in people with obesity is also associated with increased inflammation. Supplementation with the *L. rhamnosus* strain may reduce the incidence and multiplicity of colon cancer precisely by inducing cell apoptosis and inhibiting inflammation [112]. Co-supplementation of *L. rhamnosus* and *L. acidophilus* has cumulated in an antitumor effect by reducing abnormal foci of intestinal mucosal crypts in rats [113].

Supplementation with *L. acidophilus*, *L. casei*, and *L. plantarum* can also affect the synthesis of linoleic acid. These have a strong anti-proliferative effect and act on colonocytes [114]. Treatment with a mixture of probiotics (*L. plantarum*, *L. acidophilus*, and *B. longum*) in patients with colorectal cancer increased the amount of cell-binding proteins (claudin, occludin, and JAM-1) and their distribution in the colorectal epithelium [115].

## 5. Diet and Colorectal Cancer

### 5.1. Diet Predisposing to Obesity, Inflammation, and Colorectal Cancer

Current scientific knowledge points to a link between excessive consumption of saturated fat and animal protein, a low supply of fiber, and a higher risk of developing colorectal cancer [116]. The Western diet is rich in red and processed meats, refined grains, soda, and sweets and low in fruits, vegetables, and whole-grain products [117].

Researchers indicate that Western dietary patterns, in particular, increase the risk of distal colon and rectal tumors. Moreover, the Western diet seems to have a greater influence on the development of tumors that are KRAS wildtype, BRAF wildtype, have no or a low CpG island methylator phenotype, and have microsatellite stability [118].

In addition to the obvious link between the Western lifestyle—potentiated by excess calorie consumption and physical inactivity—and the currently observed epidemy of obesity, the key intermediary in the relationship between diet and colorectal cancer seems to be the intestinal microbiota [116,117,119].

In the lumen of the large intestine of consumers of the Western diet, instead of fiber fermentation with the production of anti-inflammatory, antiproliferative, and immunomodulatory butyrate, there are dominant processes of protein fermentation and bile acid deconjugation into metabolites such as secondary bile acids and hydrogen sulfide, which directly contribute to the destruction of colonocytes or to barrier dysfunction, as well as epithelial permeability, inflammatory, DNA damage, and genotoxic ways, thus increasing the risk of colorectal cancer [116,119].

The Western diet is pro-inflammatory, and Shivappa et al., in a meta-analysis, observed that a Dietary Inflammatory Index (DDI) of food that is potentially proinflammatory increases the risk of CRC [120]. Similar conclusions were reached by Fan et al. in a meta-analysis of eight studies covering 880,380 participants. The authors observed that a proinflammatory diet is independently associated with an increased CRC risk [121].

Scientific research directly points to the role of red and processed meat in the development of colorectal cancer. According to the European Prospective Investigation into Cancer and Nutrition (EPIC) in more than 500,000 individuals, habitual meat consumers had a 20% higher risk of developing CRC compared to non-consumers or occasional consumers of meat [122]. Similarly, in the population-based Norwegian Women and Cancer cohort of 84.538 women, a high processed meat intake of more than 60 g processed meat a day was associated with an increased risk of the proximal colon, distal colon, and rectal cancer compared to consumption of less than 15 g of processed meat a day [123].

Potential mechanisms that may explain the negative effects of red and processed meat include: (1) excess heme iron promoting the growth of mucin-degrading bacteria, e.g., *Akkermansia muciniphila*; (2) increased production of secondary bile acids and the promotion of carcinogenesis through increased oxidative stress and regulation of the host metabolism; (3) excess metabolism of hydrogen sulfide by sulfur-reducing bacteria from inorganic sulfur used as a preservative in processed meat and amino acid-derived sulfur from red meat; and the promotion of carcinogenesis through DNA damage, impaired colonocyte nutrition, reduced integrity of the mucus layer, induction of epithelial hyperproliferation, and increased inflammation [117,124].

In the area of diet correlating with sulfur-metabolizing bacteria in the gut and colorectal cancer risk, a very interesting study was performed among 214,797 US health professionals free of IBD and cancer at baseline. The authors observed that food groups positively correlated with the abundance of the sulfur-metabolizing bacteria in the gut, including low-calorie beverages, french fries, red meats, and processed meats. Such a microbial sulfur diet, which was also characterized by the low intake of fruits, whole grains, and vegetables, positively correlated with a higher risk of CRC [125]. These outcomes are consistent with data from another analysis of data collected from 51.529 men, in which the authors observed that long-term adherence to a dietary pattern associated with sulfur-metabolizing bacteria in stool increased the risk of distal CRC [126].

Among the factors that also increase the risk of colorectal cancer is alcohol consumption. A meta-analysis of six cohort studies showed that this was the case even with small amounts of alcohol consumption. Compared with nondrinking or occasional alcohol drinking, very light (≤0.5 drink/day) or light (≤1 drink/day) drinking increased the incidence of male colorectal cancer by 6%. Potential mechanisms explaining this correlation include acetaldehyde production in the colon, cell proliferation due to ethanol or acetaldehyde exposure, and alterations in DNA repair mechanisms [127]. Furthermore, in a meta-analysis of epidemiological studies, there was a modest positive association between heavy alcohol drinking (>50 g/day of ethanol) and CRC mortality [128].

### 5.2. Diet Protecting against the Development of Obesity, Inflammation, and Colorectal Cancer

The Mediterranean diet (MD) is a dietary pattern typical for the population living in the Mediterranean Basin. The MD is characterized by a high intake of plant foods (e.g., vegetables, fruits, and grains), a moderate intake of dairy products, fish, and eggs, and a low intake of red meat and sweets [129]. According to the study, a high adherence to the MD protects against colorectal cancer, especially distal colon cancer in men [130]. According to Acevedo-León, MD increased the level of glutathione peroxidase. Additionally, this diet decreased 8-oxoDG (8-oxo-7′8-dihydro-2′-deoxyguanosine), which is one of the oxidized DNA bases in colorectal cancer patients [131]. Additionally, the Mediterranean diet contains many components that reduce the risk of colorectal cancer, e.g., fruits and vegetables, which contain fiber that protects against colorectal cancer [132]. Moreover, the Mediterranean diet is rich in fish, the consumption of which is also inversely associated with the risk of colorectal cancer [133]. An American study showed that a high intake of vegetables, fruits, and fat-reduced foods and a low frequency of meat and potato consumption reduced the risk of colorectal cancer [134]. Moreover, phenolic compounds of olive oil reduce proliferation, migration, invasion, and angiogenesis due to regulating numerous signaling pathways [135]. Additionally, the MD decreases gut microbiota and strengthens the immune system, decreasing inflammation [136].

The systematic review showed that vitamin D protects against colorectal cancer, and the Mediterranean diet was the only dietary pattern that may prevent this type of cancer. Surprisingly, vegetarians, whose diet is rich in fruits and vegetables, present a higher risk of colorectal cancer than meat eaters [137]. On the other hand, Wu et al. reported that plant-based diets are associated with a decreased risk of colorectal cancer; however, the risk depends on the quality of the diet [138].

According to the American Institute for Cancer Research, there is strong evidence that whole grains, food containing fiber, calcium supplements, and dairy products protect against colorectal cancer [41]. A meta-analysis showed that the risk of colorectal cancer is decreased by 17% for each 90 g/day increase in whole grain [133]. In fact, fiber consumption was inversely correlated with CRP [139], so it probably affects inflammation. Whole-grain food consumption also decreases at least one level of inflammatory markers [140]. Additionally, Harland and Garton reported that whole-grain consumption was associated with lower BMI and visceral adiposity [141]. In turn, many dairy products contain probiotic bacteria that modulate the gut microbiota composition, affecting the immune system [142]. Additionally, a high-dairy diet affects the expression of genes related to inflammation pathways [143]. Vieira et al. in a meta-analysis, reported that the risk of colorectal cancer is decreased by 13% for each 400 g/d increase in dairy products [133].

Additionally, body fat increases the risk of colorectal cancer. However, there has been no study referring to the reduction in colorectal risk after body weight loss [144]. Bariatric surgery decreased the risk of breast and endometrial cancer, but there was no association between bariatric surgery and other types of cancer, including colorectal cancer [145]. However, physical activity, which is part of obesity therapy, decreases the risk of colorectal cancer [146].

In conclusion, the Mediterranean diet and the high consumption of vegetables, fruits, whole grains, and dairy products may affect the body mass, gut microbiota, and immune system. Therefore, these practices decrease inflammation and reduce the risk of colorectal cancer.

A summary of dietary factors and the risk of CRC is presented in Table 2.

## 6. Sedentary Lifestyle

Regular physical activity presents a preventive role for CRC development and decreases the risk of CRC by 12% to 28%, depending on the level of physical activity [148]. Moreover, the mortality and recurrence rates in patients after diagnosis are lower in physically active patients than in patients lacking regular physical activity, highlighting its role in tertiary prevention [149]. Mechanisms explaining this association include reductions in circulating insulin and IGF-1 and proinflammatory cytokines and leptin through the reduction of adiposity [150,151] and the regulation of DNA damage and repair [148].

Sedentary behaviors are defined as activities with energy expenditures of ≤1.5 the metabolic equivalents of tasks (METs) performed in the sitting, reclining, or lying postures, e.g., watching television, reading a book, or working at a computer [151].

According to the 2018 PAGA Committee Scientific Report, there is moderate evidence that sedentary behavior is associated with an increased risk of colon cancer [152].

## 7. Summary and Conclusions

**Figure 1:**
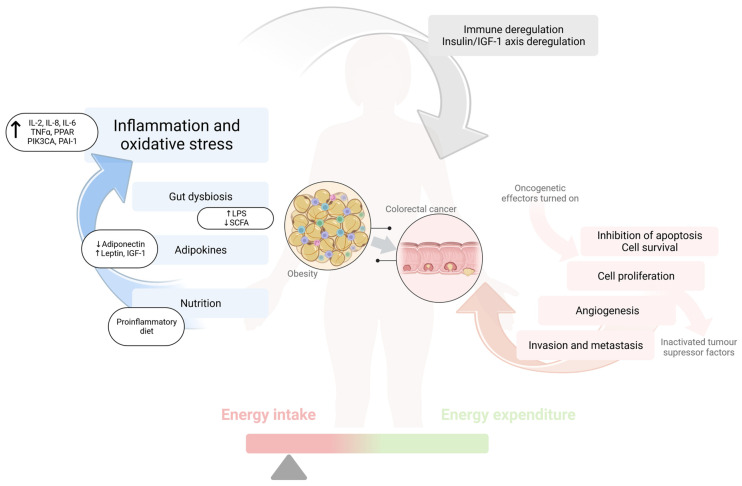
Obesity-related factors and CRC risk.

**Figure 2:**
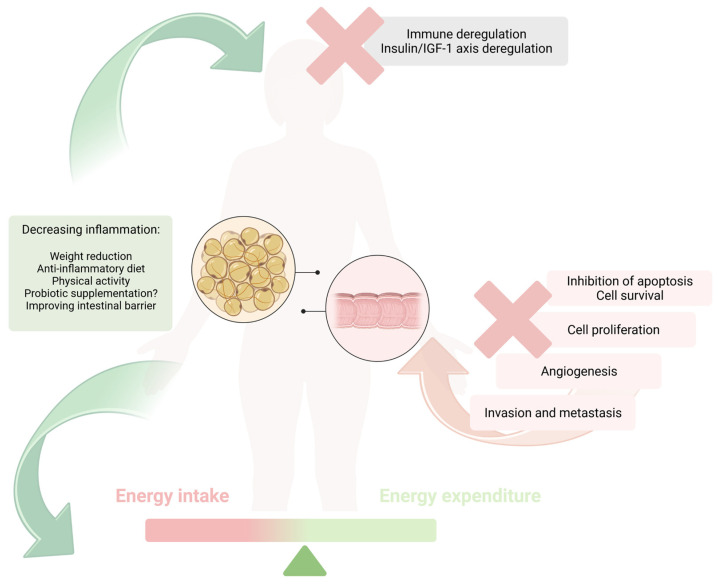
Summarized approaches focused on obesity-related factors increasing the risk of CRC.

Obesity is associated with low-grade inflammation, an unfavorable adipokine profile, macrophage recruitment, and oxidative stress, increasing the risk of colorectal cancer (Figure 1). However, as we have discussed in our paper, many other obesity-related factors, e.g., an unhealthy diet and gut microbiota dysbiosis, are undoubtedly associated with an increased risk of colorectal cancer. Taking the current data presented in the manuscript into account, the question we should be asking is not about the (at this moment obvious) correlation between obesity and diet, but rather how we should modify the current approach to the treatment of obesity in order to decrease obesity-related cancer risk more significantly (Figure 2). Although in the case of proper dietary behaviors or physical activity, the case seems more clear, with regard to other, no less essential aspects, more studies are definitely needed. An anti-inflammatory diet, including a high intake of polyphenols (e.g., fresh fruits and vegetables, high-quality vegetable and fish oils), fiber, and dairy products, along with a decreased consumption of red and processed meat, alcohol, and simple sugars, will be essential in mitigating both obesity and CRC risk. Moreover, behavioral and clinical treatments should focus on weight reduction, not only because of the beneficial effect on metabolic health, but also in order to decrease obesity-related increased risk of cancerogenesis and, moreover, to improve tertiary prevention. Further, the treatment of gut dysbiosis, which is frequently present among individuals with obesity, seems to be intriguing aspect, possibly decreasing CRC risk, and providing a new insight into primary prevention. Nevertheless, more RCTs are needed to provide direct guidelines regarding the type and duration of probiotic supplementation (with *Lactobacillus* as a potentially beneficial strain). The microbiota-centered approach should include dietary modifications in the context of improving intestinal integrity.

## Figures and Tables

**Table 1:** Obesity prevalence among CRC cases and its relation with chosen CRC outcome.

Population (*n*, Total)	Years	CRC Cases, *n*	Individuals with Obesity and CRC *, %	Individuals with Obesity *, *n*	CRC Risk OR/RR/HZ (CI)	Ref.
Australia, men (16,556)	1990–1994	153	33.33	51 (BMI > 29.2)	CRC risk RR = 2.1 (1.3–3.7) 4th vs. 1st quartiles of WHR	[56]
United Kingdom (40,467)	2006–2010	1918	28.94	555	n/a	[59]
Germany (14,552)	2003–2020	747	16.2	121 (10 years before diagnosis)	CRC risk OR = 2.17 (1.54–3.07)	[60]
Singapur (51,251)	1993–1998	980	11.63	114 (BMI > 27.5)	CRC risk HR = 1.25 (1.01–1.55) Colon cancer HR = 1.48 (1.13–1.92) Rectal cancer HR = 0.93 (0.64–1.36)	[61]
Japan, men Cohort I (16,765) Cohort II (28,945)	1990–2001	-	-	420 616	CRC risk RR = 1.5 (0.7–3.0) RR = 1.5 (0.6–3.03)	[62]
Japan, women Cohort I (21,725) Cohort II (32,066)	1990–2001	-	-	700 1009	CRC risk RR = 0.7 (0.3–2.0) RR = 0.8 (0.3–2.0)	[62]
Japan, women (15,054)	1984–1992	115	7.8%	9	CRC risk RR = 2.06 (1.03–4.13)	[63]
The USA (517,144)	1995–2000	3343	25.04	837	Colon cancer risk M/W BMI 30–32.5 M, RR = 1.53 (1.23–1.9) W, RR =1.28 (0.97–1.69) BMI ≥ 40 M, RR = 2.39 (1.59–3.58)/ W, RR = 1.49 (0.98 = 2.25)	[64]
The USA (36,941)	1986–2005	1464	27.46	402	CRC risk RR = 1.56 (1.10–2.22)	[65]

* BMI ≥ 30 kg/m^2^. CRC—colorectal cancer; OR—odds ratio; RR—relative risk; HZ—hazard-ratio; unless otherwise stated. M—men; W—women; n/a—not applicable; OR—odds ratio; RR—relative risk; HR—hazard ratio; CI—confidence interval.

**Table 2:** Foods and dietary patterns increasing or decreasing the risk of colorectal cancer (dietary patterns are characterized in the text).

Foods and Dietary Patterns Increasing the Risk of Colorectal Cancer	Foods and Dietary Patterns Decreasing the Risk of Colorectal Cancer
Western diet [118] Sulfur microbial diet [125,126] Proinflammatory diet [120,121] Red and processed meat [122,123] Refined grains [147] Alcohol [127]	Mediterranean diet [130,137] Fruits and vegetables [132,134] Fiber [41,132] Phenolic of olive oils [135] Fish [133] Vitamin D [137] Whole grains [41]. Dairy products [41,133]

## Data Availability

Data are available in a publicly accessible. The data presented in this study are openly available in Medline and PubMed databases and on the publisher’s website. The keywords that were used: colorectal cancer; diet; obesity; inflammation; cytokines; microbiota. All data in the text are quoted and all works used are listed in the bibliography along with doi and reference numbers.

## Abbreviations

CRC	colorectal cancer
WHO	World Health Organization
IARC	International Agency for Research on Cancer
IBD	inflammatory bowel disease
IGF-1	insulin-like growth factor (IGF)-1
IGF-1R	IGF-1 receptor
IL	interleukin
TNF	tumor necrosis factor
WHR	waist-to-hip ratio
WC	waist circumference
HR	hazard ratio
RR	relative risk
LPS	lipopolysaccharide
BCAA	branched-chain amino acid
SCFAs	short-chain fatty acids
TLR-4	toll-like receptor 4
